# Unveiling hypoxic regulatory networks by bioinformatics: mechanisms of hypoxia-related hub genes driving rituximab resistance and poor prognosis in DLBCL

**DOI:** 10.3389/fonc.2025.1592441

**Published:** 2025-10-20

**Authors:** Jiayi Yao, Juan Huang, Yuanfei Mao, Zizhen Xu

**Affiliations:** ^1^ Department of Laboratory Medicine, College of Health Science and Technology, Ruijin Hospital, Shanghai Jiao Tong University School of Medicine, Shanghai, China; ^2^ Department of Clinical Laboratory, Yangpu Hospital Affiliated to Tongji University, Shanghai, China; ^3^ State Key Laboratory of Medical Genomics, Department of Hematology, Ruijin Hospital, Shanghai Jiao Tong University School of Medicine, Shanghai Institute of Hematology, Shanghai, China; ^4^ State Key Laboratory of Translational Medicine (Shanghai), Department of Hematology, Ruijin Hospital, Shanghai Jiao Tong University School of Medicine, Shanghai Institute of Hematology, Shanghai, China

**Keywords:** diffuse large B-cell lymphoma, hypoxia, rituximab, drug resistance, B cell receptor signaling pathway, bioinformatics

## Abstract

**Background:**

Diffuse large B-cell lymphoma (DLBCL), an aggressive subtype of non-Hodgkin lymphoma, exhibits heterogeneous clinical outcomes. While rituximab, a CD20 inhibitor, combined with chemotherapy has improved survival in some patients, resistance remains prevalent, particularly in hypoxic tumor microenvironments. Understanding hypoxia-related genes (HRGs) and their role in rituximab resistance is critical to addressing therapeutic challenges in high-risk DLBCL.

**Methods:**

Gene expression profiles from GEO datasets (GSE56315: DLBCL tumor vs. normal; GSE104212: hypoxia-treated DLBCL cell lines) were analyzed to identify overlapping genes between DLBCL-signature genes (DSGs) and HRGs. protein interaction network topology analysis and Lasso regression modeling of TCGA-DLBC dataset were employed to screen regulator and hub genes. Hub genes linked to rituximab response and survival were validated in DLBCL patients receiving rituximab therapy. Functional enrichment analysis was used to explore associated pathways. The expression of the identified regulator and hub genes was validated using reverse transcription quantitative polymerase chain reaction (RT-qPCR).

**Results:**

58 overlapping genes were identified between DSGs and HRGs. PPI network and Lasso regression revealed 5 *MS4A1* regulator genes and 10 hub genes. Among these, *LGALS1* (HR = 0.588, *p* = 0.00085), *TIMP1* (HR = 0.591, *p* = 0.00098), *ANXA1* (HR = 0.614, p=0.0024) and *STAP1* (HR = 0.633, p=0.0035) were significantly associated with overall survival and *GPNMB* (AUC = 0.869), *CDCA7* (AUC = 0.686), and *STAP1* (AUC = 0.663) associated with treatment response in rituximab-treated patients. Functional analysis implicated these genes in B-cell receptor (BCR) and PI3K-AKT signaling pathways, suggesting their mechanistic roles in therapeutic resistance.

**Conclusions:**

This study identifies hypoxia-associated genes critical to rituximab resistance in DLBCL, highlighting potential therapeutic targets. Their involvement in BCR and PI3K-AKT pathways underscores novel vulnerabilities for overcoming refractory disease. Our findings provide a foundation for developing strategies to improve outcomes in high-risk DLBCL patients with hypoxic microenvironments.

## 1 Introduction

Diffuse large B-cell lymphoma (DLBCL) is the most prevalent type of non-Hodgkin lymphoma (NHL) accounting for 30–40%, which has an extensive effect on patient survival and quality of life (1). DLBCL are aggressive malignancies with relatively mature treatment programs. Current treatments for DLBCL include chemotherapy, immunotherapy, and in some cases, stem cell transplantation. The first-line standard of care for patients is R-CHOP therapy (rituximab, cyclophosphamide, doxorubicin, vincristine, and prednisone) (2–4). While R-CHOP achieves durable remission in 60-70% of newly diagnosed patients, many of them face poor prognosis, rapid progress, with over 40% of patients developing refractory disease (5). A significant number of patients develop resistance to the therapy, leading to limited treatment options (6).

Rituximab, a monoclonal antibody targeting CD20 (encoded by *MS4A1*) in B cells, has been a cornerstone of DLBCL treatment (7, 8). The mechanism is that CD20 is highly expressed on microvilli in conjunction with monoclonal antibodies, leading to antibody concentration-dependent B-cell polarization and stabilization of microvillus protrusions, and killing of tumor B-cells through antibody-dependent cytotoxicity (ADCC) and complement-dependent cytotoxicity (CDC) (9). Although the results for DLBCL patients have greatly improved, the emergence of resistance remains a major clinical challenge. Currently, the prevailing resistance mechanisms for rituximab treatment of DLBCL are divided into endogenous and exogenous mechanisms, with the endogenous pathway including inhibition of intracellular drug transport, inhibition of drug activation, and increased drug degradation and efflux. Exogenous pathways include hypoxia, acidosis and extracellular matrix alterations (6).

Among the resistance drivers above, hypoxia emerges as a critical microenvironmental stressor that may subvert rituximab’s efficacy through multiple axes. Hypoxia is a hallmark of aggressive DLBCL, which helps lymphoma cells adapt to long-term hypoxia microenvironment by upregulating proteins involved in glucose utilization, degrading mitochondrial proteins for potential mitochondrial recycling, and becoming more reliant on BCL-2 and PI3K-AKT-mTOR signaling for survival (10).

Hypoxic stress profoundly remodels molecular landscapes in Non-Hodgkin B-cell malignancies, triggering adaptive modifications in key mediators of tumor survival such as glucose transporter 1 (GLUT-1/SLC2A1), carbonic anhydrase isoforms (CAIX/CA9 and CAXII/CA12), and vascular endothelial growth factor (VEGF) (11). Besides, it plays an essential role in angiogenesis, regulate glucose metabolism, and control cancer cell invasion and metastasis (12). These hypoxia-induced alterations drive metabolic reprogramming and angiogenesis during malignant progression.

Variable numbers of immune system cells, stromal cells, blood vessels, and extracellular matrix components constitute the microenvironment around B cell lymphomas (13). While hypoxia-induced metabolic remodeling has been well characterized in solid tumors, its functional consequences in DLBCL microenvironments, particularly regarding therapeutic resistance, remain largely unexplored (14).

Understanding the molecular mechanisms underlying hypoxia-induced rituximab resistance in DLBCL is essential for developing viable and efficient therapeutic strategies. In our research, we aim to identify important genes and associated pathways involved in hypoxia-induced rituximab resistance in DLBCL with bioinformatics tools, providing new insights into the mechanisms of resistance and potential therapeutic targets.

## 2 Materials and methods

To identify differential expression genes induced by hypoxia in DLBCL, two datasets (GSE56315 (15) and GSE104212 (12)) were retrieved from the GEO database and analyzed. Protein–protein interaction (PPI) network construction and functional enrichment analysis including Gene Ontology (GO), Kyoto Encyclopedia of Genes and Genomes (KEGG) and gene set enrichment analysis (GSEA) was then performed to screen for hub genes among the differential expression genes. Least absolute Shrinkage and Selection Operator (LASSO) regression analysis models were developed based on the expression of common differential expression genes in the TCGA database to identify genes with a strong association with *MS4A1* expression (16).

**Figure 1:**
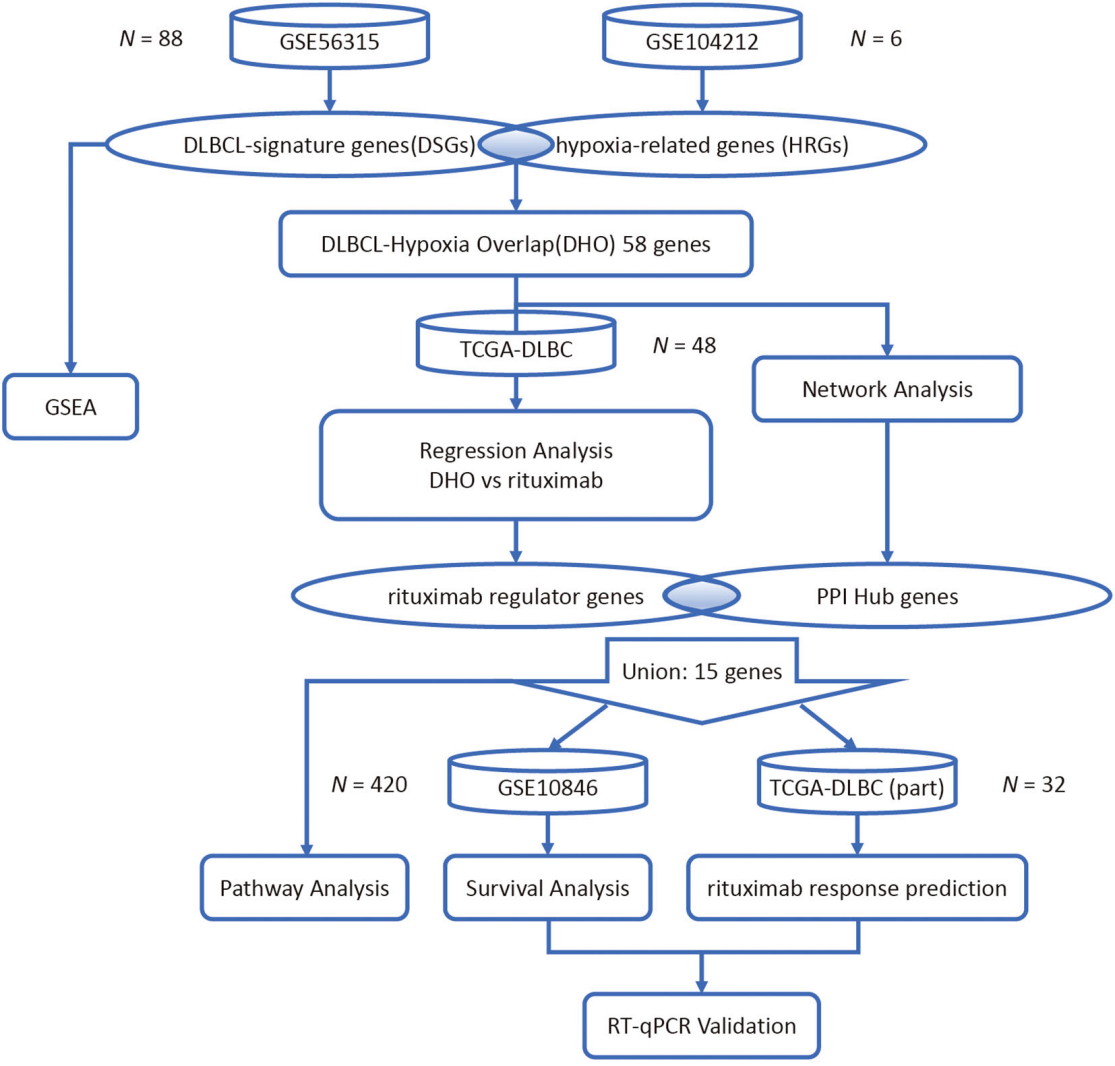
Research flowchart.

In order to determine potential signaling pathways regulating *MS4A1* expression (which indicates the expression level of CD20) in the hypoxic DLBCL tumor microenvironment, we employed the Kaplan-Meier survival analysis to assess the survival and response rates of PPI hub genes after rituximab treatment. To validate the expression of the regulator genes and the hub genes in DLBCL, we lastly conducted the reverse transcription quantitative polymerase chain reaction (RT-qPCR) analysis on the 6 genes with clinical significance in patients’ survival and rituximab treatment response in 4 cell lines (**Figure 1**).

### 2.1 Gene expression data collection and processing

GSE10846 (17), GSE56315 and GSE104212 were all obtained from the NCBI Gene Expression Omnibus (GEO) public database. The first two datasets were annotated with the GPL570 platform, while the latter was annotated with GPL10558. In the GSE10846 dataset, a total of 412 DLBCL patients with complete expression profiles and corresponding survival data were included. In the GSE56315 dataset, the gene expression levels of 55 tumor and 33 normal samples from DLBCL patients were profiled using a single-channel array platform. In the GSE104212 dataset, SUDHL2 cell line samples were subjected to hypoxic conditions (1% O_2_) and normoxic controls (21% O_2_). The Illumina platform was used to profile the gene expression levels of 6 related samples, with 3 biological replicates conducted for each microenvironment condition.

An expression matrix including distinct gene annotations was then created by averaging the expression levels of each gene that was probed by respective probes on the microarray chip. The datasets GSE10846, GSE56315 and GSE104212 have already undergone Variance Stabilizing Normalization (VSN), hence there is no need to apply a log2-transformation to them. All the matrices underwent standardization to achieve normally distributed expression levels.

### 2.2 Identification of differential expression genes

Transcriptome-wide differential expression analysis was systematically conducted according to fold change (FC) and t-test. For the GSE56315 dataset, paired-samples t-test was implemented to quantify the gene expression difference between the tumor and normal samples. DLBCL-signature genes (DSGs) were defined as differential expression genes exhibiting both statistical significance (adjusted *p*-value < 0.05) and biological effect sizes, specifically requiring FC > 8.101 for upregulated genes and < 0.135 for downregulated genes in tumor samples relative to matched controls. Hypoxia-related genes (HRGs) within the GSE104212 dataset were identified using FC cutoffs > 2.186 for upregulation and < 0.444 for downregulation, with analogous statistical criteria (adjusted *p*-value < 0.05), reflecting dataset-specific dynamic ranges. The FC thresholds were established according to the number of upregulated and downregulated genes requiring selection. Intersectional analysis of DSGs and HRGs was performed using the “ggvenn” R package (v0.1.9), generating a consensus gene set designated as DLBCL-hypoxia overlap (DHO) genes, which underwent subsequent functional characterization.

### 2.3 Functional profiling and pathway enrichment analysis

To elucidate the biological properties of DSGs and HRGs, a tiered functional annotation strategy was employed. Primary annotation utilized the GO database (http://geneontology.org, on December 3^rd^, 2024), systematically categorizing genes into three domains: biological processes (BP, involves the biological activities the gene participates in), cellular components (CC, refers to the specific location of a gene product in the cell), and molecular functions (MF, specifies the gene molecular level capabilities). Enrichment significance was computed with FDR correction (*p* value < 0.05). Subsequently, pathway enrichment results were achieved through KEGG database (https://www.kegg.jp/, on January 28^th^, 2025), identifying potential signaling networks and retaining pathways with both statistical significance (*p* value < 0.05) and ≥ 10 constituent genes from the target sets. To dissect hypoxia-associated pathway perturbations beyond individual gene effects, GSEA was executed using a non-parametric computational framework. The analysis incorporated 1,000 phenotype-based permutations to establish empirical null distributions, thereby controlling for dataset-specific background signals. MSigDB (v7.5.1) includes 9 datasets covering more than 16,000 gene sets, among which “C2: curated gene sets” was selected for this study to discover the potential signaling pathways influenced by hypoxia mechanisms of DLBCL. Enrichment scores were calculated via the “fgsea” R package, with significance thresholds set at *p* value < 0.05. This approach enabled detection of coordinated transcriptional shifts across functionally related gene clusters, complementing single-gene differential expression findings.

### 2.4 Protein interaction network topology analysis

Protein-protein interaction (PPI) networks for DHO genes were constructed using the search tool for the retrieval of interacting genes database (STRING, http://string-db.org/, on January 26^th^, 2025), integrating experimental evidence, curated knowledge, and computational predictions. Interactions with composite confidence scores > 0.4 were retained to ensure high-confidence network architecture (18). Topological analysis was performed using cytoHubba (a Cytoscape plugin implementing graph-theoretical algorithms, v0.1). The maximum clique centrality (MCC) metric was prioritized for hub gene identification due to its stable performance in detecting functionally critical nodes within scale-free networks (19). MCC values were computed as:


$$MCC(v)\;=\Sigma_{C\in S(v)}(|C|-\;1)!$$

In the formula above, *S(v)* represents all maximal cliques which contain node *v*. The top 10 nodes by MCC score were classified as network hubs, reflecting their roles as integrative signaling coordinators.

### 2.5 Lasso regression analysis modeling

To delineate DHO genes influencing *MS4A1* (CD20) expression, RNA-seq expression data from 48 DLBCL specimens of TCGA-DLBC were downloaded from The Cancer Genome Atlas Program database (https://www.cancer.gov/ccg/research/genome-sequencing/tcga, on January 31^st^, 2025) and then standardized. LASSO regression was implemented via the “glmnet” R package, employing L1-penalized least squares minimization. Compared with other models, it produces more stable and reproducible results while inherently performing feature selection by shrinking irrelevant coefficients to zero and mitigating multicollinearity, thereby enhancing model interpretability. In order to screen the genes from DHOs that majorly affect the *MS4A1* expression level, significant regulatory associations were defined at *p* value < 0.05, the relevant formula is shown below:


$$Y\;=\;w_{0}\;+\;w_{1}x_{1}\;+\;w_{2}x_{2}\;+\;.\;.\;.\;+\;w_{n}x_{n}$$

In the formula above, Y represents the expression level of *MS4A1*. x_n_ and w_n_ respectively denotes the expression level of the nth selected gene and its corresponding coefficient, which quantifies its influence on *MS4A1* expression. These genes are designated as *MS4A1* regulator genes. The term “regulator” is used in a statistical/network sense that genes whose expression level is strongly and reproducibly associated with MS4A1 expression—without implying direct molecular control.

### 2.6 Survival analysis and drug response prediction

Progression-free survival in rituximab-treated patients from GSE10846 dataset was analyzed using the “survminer” R package. Patients were dichotomized into high or low expression groups by median gene expression pretreatment. Kaplan-Meier curves were visualized and then compared via log-rank tests, with hazard ratios (HR) and 95% confidence intervals computed through Cox proportional hazards models (20). Afterwards, treatment response predictability was assessed using receiver operating characteristic (ROC) analysis based on 32 DLBCL patients in the TCGA-DLBC datasets, with area under the curve (AUC) quantifying classification accuracy. The “pROC” R package was utilized to assess the capability of the expression levels of each of the PPI hub genes as well as the *MS4A1* regulator genes in the prediction of patients’ responses to rituximab treatment. All *p*-values were calculated by Wilcoxon rank-sum test, and the 95% confidence interval was calculated by Delong Test. Besides, KEGG pathway analysis of *MS4A1* regulators genes and hub genes were conducted via ShinyGo 0.77 platform (http://bioinformatics.sdstate.edu/go77/, February 3^rd^, 2025), applying FDR correction < 0.05 to identify potential therapeutic target pathways.

### 2.7 Cell culture

Human DLBCL cell lines SU-DHL-6 (SU6), SU-DHL-8 (SU8), RIVA (RI-1), and U-2932 were used in this study. These cell lines, gathered from the Shanghai Institute of Hematology, located at Ruijin Hospital, affiliated with the School of Medicine at Shanghai Jiao Tong University in Shanghai, China, were cultured in RPMI-1640 medium (Gibco, USA) supplemented with 10% fetal bovine serum (FBS) and 1% penicillin-streptomycin. Cells were maintained at 37°C in a humidified incubator with 5% CO_2_. For hypoxia treatment, cells were exposed to 1% O_2_, 5% CO_2_, and balanced N_2_ for 24 hours in a hypoxia chamber. Normoxia controls were maintained at 21% O_2_. Each cell line was subjected to both normoxia and hypoxia conditions, with SU-DHL-6 normoxia serving as the external calibration for relative expression analysis (relative expression = 1.0).

### 2.8 RT-qPCR

Total RNA was extracted using TRIzol reagent (Invitrogen, USA), followed by on-column DNase digestion to remove genomic DNA contamination. RNA quality was assessed by measuring the A260/280 ratio (target range: 1.8-2.1) and checking integrity via 1% agarose gel electrophoresis or Bioanalyzer. For cDNA synthesis, 1 μg of total RNA was reverse transcribed using a mix of random primers and oligo(dT), following the manufacturer’s instructions for PrimeScript RT Kit (Takara, Japan). The qPCR reaction was performed on a Roche LightCycler^®^ 480 system using ChamQ Universal SYBR qPCR Master Mix (Vazyme, Q711-02) with a primer final concentration of 0.2 μM and a 1:5 dilution of cDNA. Relative gene expression was calculated using the 2^-^ΔΔCt method, with ACTB as the housekeeping gene. The specific primer sequences employed in this process are detailed in **Table 1**.

**Table 1:** Primer sequences used for RT-qPCR analysis.

Gene	Forward primer (5'-3')	Reverse primer (5'-3')
LGALS1	AGCAGCGGGAGGCTGTCTTTC	ATCCATCTGGCAGCTTGACGGT
TIMP1	GGAGAGTGTCTGCGGATACTTC	GCAGGTAGTGATGTGCAAGAGTC
ANXA1	GCGAAACAATGCACAGCGTCAAC	CAACCTCCTCAAGGTGACCTGT
STAP1	GGAGGATTGAGACAGAGCAGAG	CTTCTGGAGCATCTCAGTTGCC
GPNMB	GTGCTCAATGGAACCTTCAGCC	AGGAATCCTACTCAGCTCCAGG
CDCA7	CCAGGCTCCGACTCACAATCAAG	GTACTTATCCTCTTCCTCCTCCTCCTC
ACTB	CATGTACGTTGCTATCCAGGC	CTCCTTAATGTCACGCACGAT

## 3 Results

### 3.1 Identification of DSGs and HRGs in DLBCL

**Figure 2:**
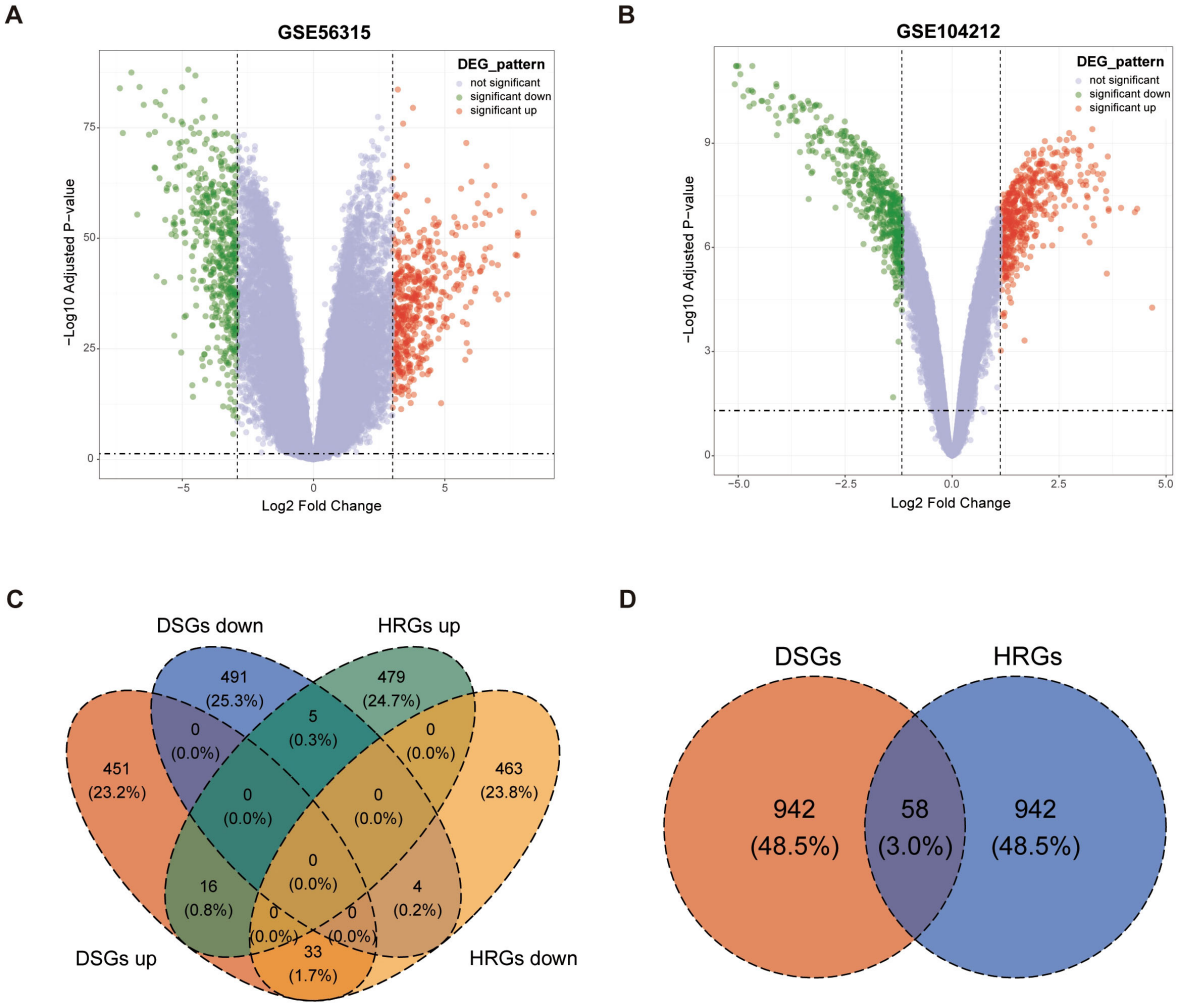
Identification of DLBCL-signature genes (DSGs) and hypoxia-related genes (HRGs): **(A)** Volcano plot for GSE56315; **(B)** Volcano plot for GSE104212; **(C)** Venn plot showing overlaps between DSGs and HRGs; **(D)** Venn plot showing overlapping gene amounts in DSGs and HRGs by the status of upregulated and downregulated.

By establishing thresholds for fold change (FC) and *p*-value, a set of 1000 DSGs were identified, consisting of 500 upregulated and 500 downregulated genes, which show differential expression in GSE56315 between tumor and paired normal tissues (**Figure 2A**). Applying the same analytical approach, another set of 1000 hypoxia-regulated genes (HRGs) were also determined, with 500 upregulated and 500 downregulated genes, reflecting the gene expression differences between hypoxic and normal microenvironment conditions in GSE104212 (**Figure 2B**). The heatmaps of DSGs and HRGs are shown in **Supplementary Figure S1**, **Supplementary Figure S2**. 58 overlapping genes, known as DLBCL-Hypoxia Overlaps (DHOs), were obtained from the intersection of DSGs and HRGs. Among these 58 genes, 21 were upregulated and 37 were downregulated in the hypoxic samples in comparison to the normal samples in GSE104212 (**Figures 2C, D**).

### 3.2 Gene set enrichment analysis of DSGs and HRGs

We performed different types of enrichment analysis of both DSGs and HRGs across multiple databases. Notably, three groups of databases yielded highly significant enrichment results with close associations to DLBCL, hypoxia, and rituximab, which deserve particular attention.

**Figure 3:**
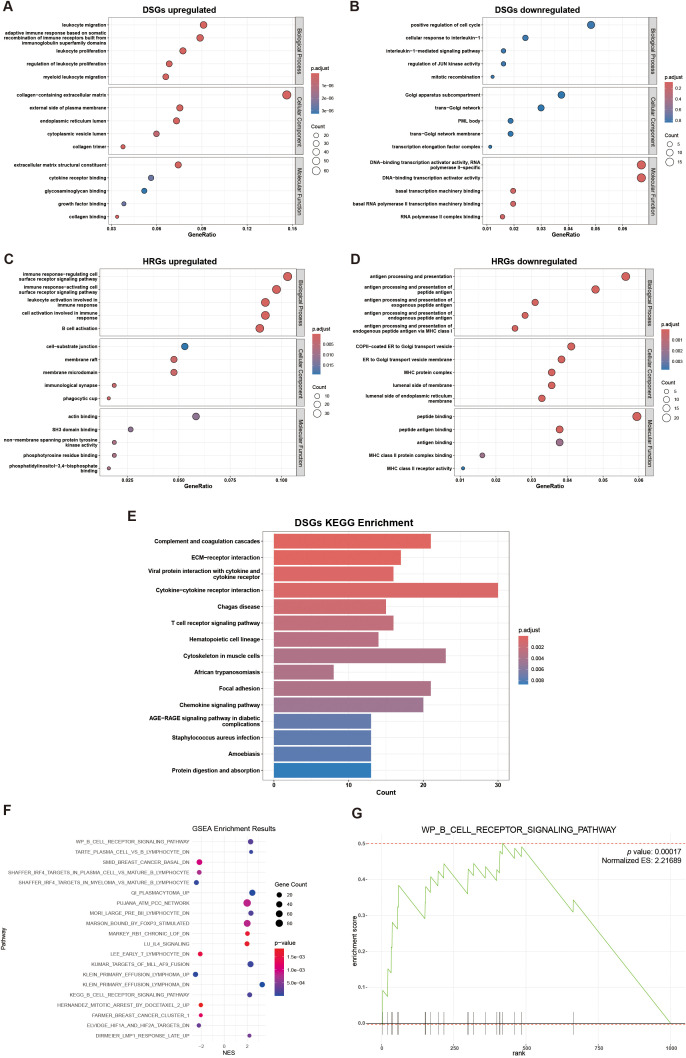
Gene set enrichment analysis: **(A)** GO enrichment analysis of DSGs upregulated; **(B)** GO enrichment analysis of DSGs downregulated (not significant); **(C)** GO enrichment analysis of HRGs upregulated; **(D)** GO enrichment analysis of HRGs downregulated; **(E)** KEGG enrichment analyses of DSGs; **(F)** Scatter plot of GSEA enrichment results of HRGs; **(G)** GSEA plot for WP_B_CELL_RECEPTOR_SIGNALING_PATHWAY, showing gene distribution and enrichment score.

In the GO database, DSGs upregulated genes were found mainly enriched in “leukocyte migration”, “collagen-containing extracellular matrix”, and “extracellular matrix structural constituent” gene sets, DSGs downregulated genes weren’t significantly enriched in any pathway. While, HRGs upregulated genes were found significantly enriched in “cell-substrate junction”, “immune response cell surface receptor” and “actin binding” pathways, and HRGs downregulated genes were mainly enriched in “antigen processing and presentation”, “COPI-coated ER to Golgi transport vesicle”, and “peptide binding” gene set (**Figures 3A-D**). In the KEGG databases, upregulated DSGs were enriched in “Complement and coagulation cascades”, “Cytokine-cytokine receptor interaction”, and “ECM-receptor interaction” pathways (**Figure 3E**). In the Molecular Signatures Database, we found HRGs were potentially enriched in “KEGG_B_CELL_RECEPTOR_SIGNALING_PATHWAY” gene set and “WP_B_CELL_RECEPTOR_SIGNALING_PATHWAY” gene set (**Figures 3F-G**).

### 3.3 Model establishment of the impact of DHOs on MS4A1 expression in the TCGA-DLBC dataset

**Figure 4:**
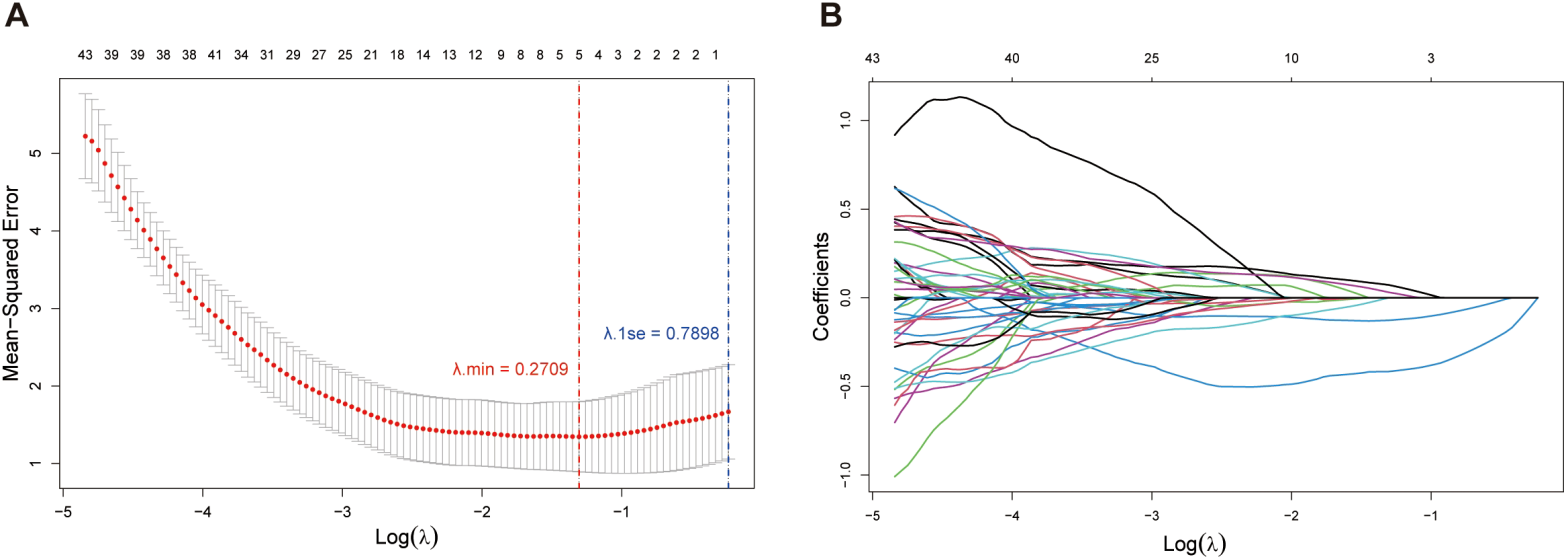
Lasso regression analysis: **(A)** Relationship between Mean-Squared Error (MSE) and the regularization parameter *λ* on a logarithmic scale. The error bars represent the variability of the MSE at each *λ* value; **(B)** Paths of coefficients for each feature in Lasso regression as the regularization parameter *λ* varies on a logarithmic scale.


In order to characterize the resistance to rituximab, we selected the gene for its drug target CD20, *MS4A1*, as the dependent variable. Through further LASSO regression analysis of the 58 DHOs, we pinpointed 5 genes (*RNF130*, *MT1E*, *TSPO*, *CDCA7*, *STAP1*) as key risk factors influencing *MS4A1* expression, which were utilized to establish the rituximab-resistance gene regulator model. Among many values of λ, we chose the minimal value to fit the model to achieve the highest fitting accuracy (**Figures 4A, B**). Lasso analysis was applied to assess how DHOs affect *MS4A1* expression. The gene regulator model is expressed as a weighted sum of the regression coefficients and the relative expression levels of *MS4A1* regulator genes, reflecting each gene’s impact on drug resistance:

MS4A1=17.673−0.459×RNF130−0.120×MT1E−0.079×TSPO+0.119×CDCA7+0.097×STAP1

All model-included *MS4A1* regulator genes significantly affect drug resistance of rituximab (*p* value < 0.05). Notably, *RNF130*, *MT1E*, and *CDCA7* have the largest coefficient magnitudes among these regulator genes, which represent a stronger correlation with *MS4A1* expression.

### 3.4 Protein–protein interaction network and hub genes selection of DHOs

**Figure 5:**
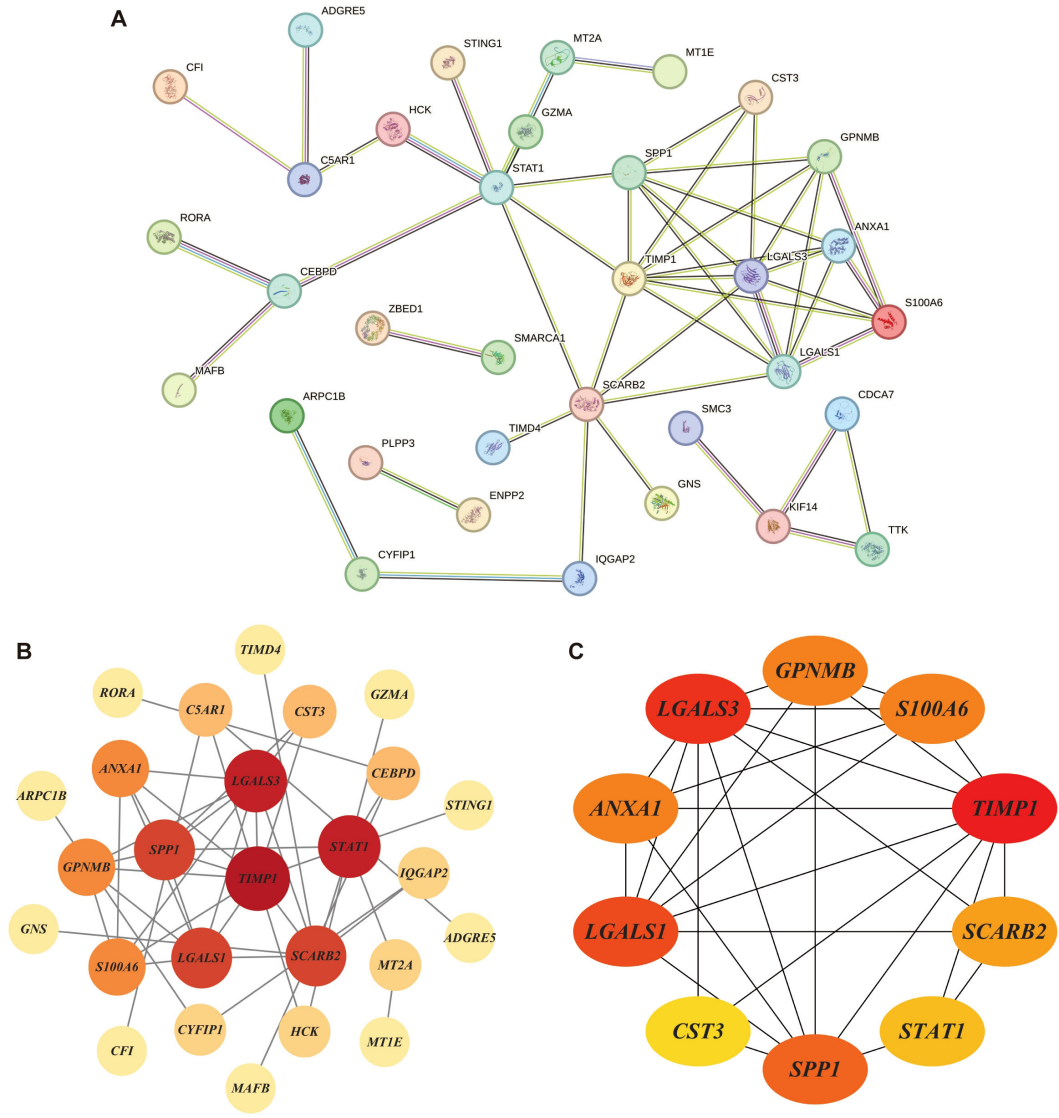
Protein–protein interaction (PPI) networks: **(A)** PPI network of DHOs; **(B)** Connections among 25 genes with confidence score > 0.4; Node size reflects the degree of connectivity, while color intensity corresponds to the combined score value; **(C)** 10 hub genes with maximum clique centrality (MCC) score > 6; Genes with a confidence score *≤* 0.4 are excluded; Darker node colors indicate higher MCC values.

The 58 DHOs were analyzed for PPI network construction by using the STRING platform. In the PPI network, by setting the confidence score threshold > 0.4, 34 genes had potential connections with at least one another gene in the network (**Figure 5A**). These results were then imported into Cytoscape (v3.10.3) to create a more quantified network. Among the 34 DHOs with medium confidence scores, 25 genes had been involved in the major network, the remaining ones were excluded from the representation (**Figure 5B**). Additionally, the maximum clique centrality (MCC) score was calculated by applying the plug-in called CytoHubba. Furthermore, 10 genes whose MCC score > 6 were defined as the hub genes: *TIMP1*, *LGALS3*, *LGALS1*, *SPP1*, *GPNMB*, *ANXA1*, *S100A6*, *SCARB2*, *STAT1*, *CST3* (**Figure 5C**).

### 3.5 Survival analysis and rituximab response prediction

**Figure 6:**
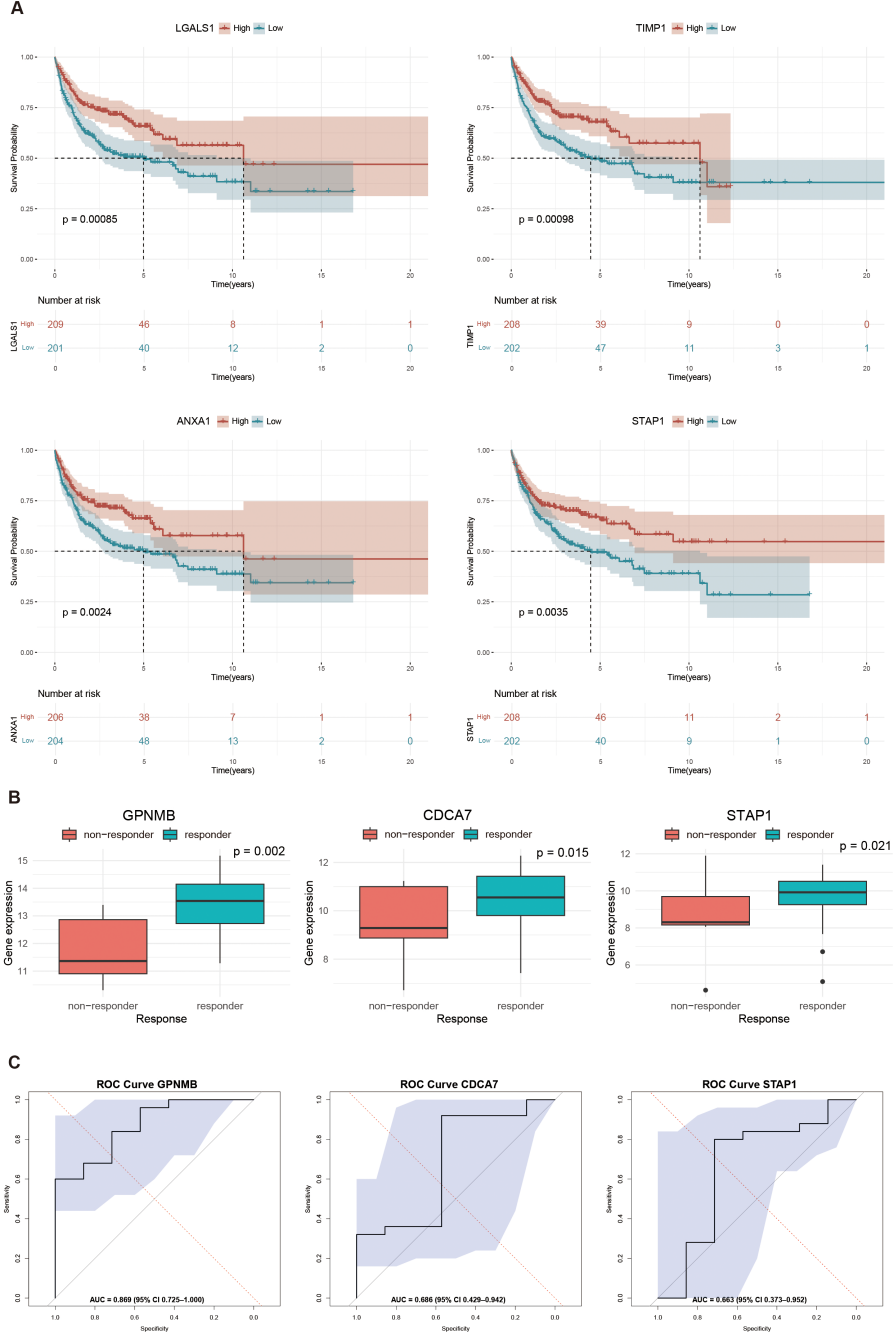
Survival analysis and response prediction: **(A)** Kaplan-Meier curves showing the survival differences between high and low expression levels of interested genes: *LGALS1* (HR = 0.588, *p* = 0.00085), *TIMP1* (HR = 0.590, *p* = 0.00098), *ANXA1* (HR = 0.614, *p* = 0.0024), *STAP1* (HR = 0.633, *p* = 0.0035); **(B)** Boxplots of top three genes in predicting rituximab response: GPNMB (*p* = 0.002), CDCA7 (*p* = 0.015), and STAP1 (*p* = 0.021); **(C)** Receiver operating characteristic (ROC) curves in predicting rituximab response: *GPNMB* (AUC = 0.869), *CDCA7* (AUC = 0.686), and *STAP1* (AUC = 0.663). Statistical significance is determined by Wilcoxon rank-sum test.

We employed the log-rank test based on clinical data of GSE10846 to compare the survival curvilinear direction for union genes, consisting of *MS4A1* regulator genes and PPI hub genes, created using the Kaplan-Meier approach. The survival analysis findings demonstrated a substantial correlation between the expression levels of 8 genes and poor prognosis of DLBCL patients. In particular, patients with higher expression levels of *LGALS1* (HR = 0.588, *p* = 0.00085), *TIMP1* (HR = 0.591, *p* = 0.00098), *ANXA1* (HR = 0.614, p=0.0024) and *STAP1* (HR = 0.633, p=0.0035) had significantly higher survival rates after rituximab treatment than those with the lower expression levels (**Figure 6A**). We further validated the gene expression of both *MS4A1* regulator genes and PPI hub genes responding to rituximab therapy. In the TCGA-DLBC cohort, the three genes with the best performance were *GPNMB* (AUC = 0.869), *CDCA7* (AUC = 0.686), and *STAP1* (AUC = 0.663) (**Figures 6B, C**).

### 3.6 KEGG pathway enrichment of union genes

**Figure 7:**
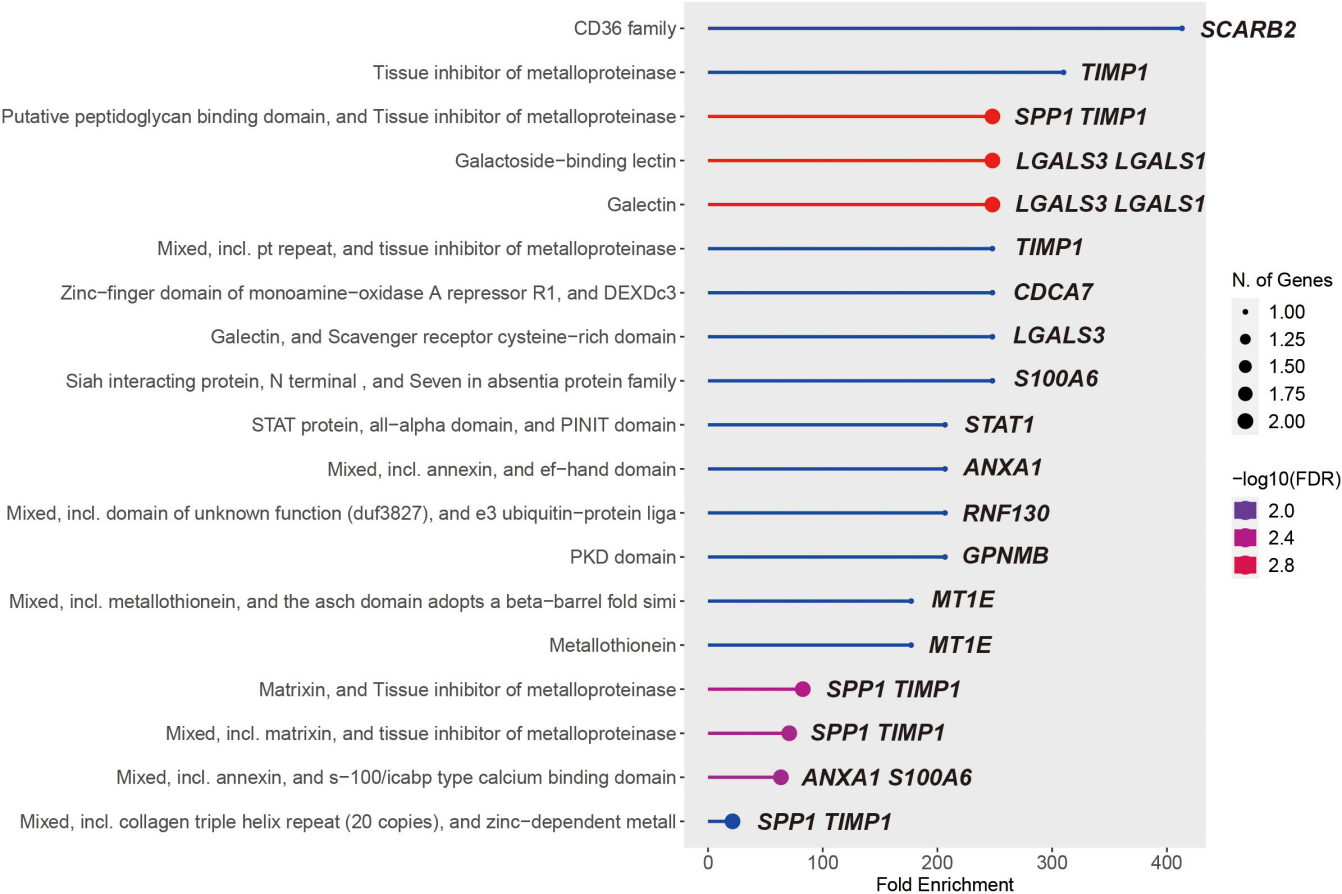
19 significantly KEGG enriched pathways of the union of *MS4A1* regulator genes and PPI hub genes.

We then performed the KEGG pathway enrichment analysis of *MS4A1* regulator genes and PPI hub genes with help of the ShinyGo platform. Over 15 significantly enriched pathways (FDRs < 0.019) were identified in different databases, most of which are associated with tissue inhibitors of metalloproteinases, immune cell surface antigens and galectins (**Figure 7**), which may affect the efficacy of rituximab by modulating immune cell function.

### 3.7 RT-qPCR validation of selected DHOs

**Figure 8:**
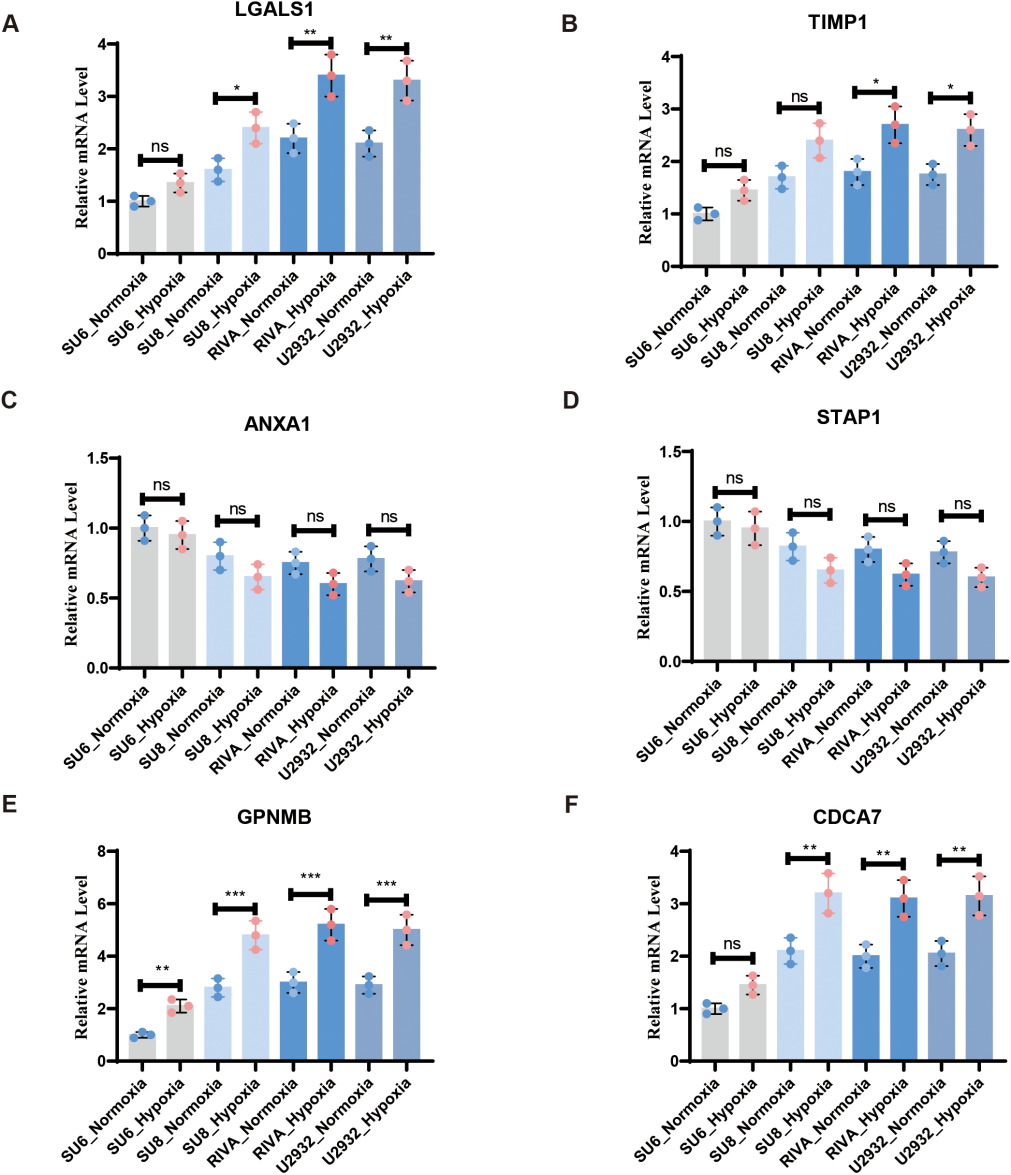
RT-qPCR confirmed the expression of DHOs in real world under hypoxia and normoxia treatment: **(A)** RT-qPCR validation of *LGALS1 in vitro* DLBCL cell lines; **(B)** RT-qPCR validation of *TIMP1 in vitro* DLBCL cell lines; **(C)** RT-qPCR validation of *ANXA1 in vitro* DLBCL cell lines; **(D)** RT-qPCR validation of *STAP1 in vitro* DLBCL cell lines; **(E)** RT-qPCR validation of *GPNMB in vitro* DLBCL cell lines; **(F)** RT-qPCR validation of *CDCA7 in vitro* DLBCL cell lines. ns, not significant; * = p < 0.05, ** = p < 0.01, *** = p < 0.001.

To examine the expression of the DHOs with clinical significance in patients’ survival and rituximab treatment response and verify their hypoxia correlation, we assessed the expression of 6 DHOs in 4 cell lines subjected to hypoxic and normoxic conditions via RT-qPCR. The results showed that *GPNMB* showed hypoxic response in all four cell lines, as evidenced by a higher relative expression under hypoxic environment. While *LGALS1*, *CDCA7* and *TIMP1* showed hypoxic response in part of DLBCL cell lines. *ANXA1* and *STAP1*, on the other hand, did not exhibit the hypoxic response in any of the four cell lines (**Figure 8A-F**).

## 4 Discussion

There is growing evidence that hypoxia significantly influences DLBCL, as it is a defining feature of malignant tumors. Hypoxia not only drives carcinogenesis but also presents a major hurdle for the proliferation of immunotherapy such as CD20 and PD-1/PD-L1 inhibitors. Thus, it is imperative to identify DLBCL biomarkers linked to and resistance to rituximab induced by hypoxia, and to clarify the relationship between them. In this study, we have identified 58 Overlapping genes (DHOs) in the GEO dataset, which represent the intersection of DSGs and HRGs. These genes may provide crucial insights into the mechanisms underlying hypoxia-driven DLBCL progression and rituximab resistance. In GSEA results, we found that the characterized genes screened were correlated with the drug target of rituximab. In particular, some of the HRGs were significantly enriched in the B-cell receptor pathway, which set the stage for subsequent analysis.

In addition, we screened 5 *MS4A1* regulator genes from DHOs by LASSO regression analysis based on TCGA-DLBC dataset. Subsequently, we identified 10 hub genes within the PPI network of DHOs. In future studies, we plan to conduct validated experiments on these genes and CD20. Based on clinical data, we conducted a profound analysis of the survival outcomes and therapy responses of *MS4A1* regulator genes and PPI hub genes following rituximab treatment, in order to confirm our analytical results. Our analysis revealed that *LGALS1*, *TIMP1*, *ANXA1*, and *STAP1* were significantly associated with treatment outcomes. These statistics highlight the critical role of *LGALS1* and *STAP1* in regulating *MS4A1* expression, thereby influencing the effectiveness of rituximab treatment.

In previous studies, rituximab is thought to inhibit B-cell survival and proliferation through negative regulation of canonical signaling pathways involving PI3K-AKT-mTOR, ERK, and mammalian target of rapamycin (21–23). And it’s also associated with down regulation of BCR immunoglobulin expression (24). As an essential drug target of rituximab, surface protein CD20 acts as a key medium of immunotherapy in various B-cell malignancies in B cell receptor signaling pathway, which turned out to be a limiting factor for inhibiting BCR activation. The genes selected in this study have numerous roles related to the key pathways mentioned above.

Galectin-1 (the product encoded by *LGALS1*) is a key ligand of the pre-B cell receptor in stromal cells, mediating the synapse formation between pre-B cells and stromal cells, as well as the triggering of pre-BCR signaling, thereby participating in the regulation of the B cell development microenvironment (25, 26). Studies have shown that *LGALS1* is significantly overexpressed in the tumor microenvironment of DLBCL, and its high expression is closely related to resistance to CD20 monoclonal antibody therapy (26). Mechanistically, *LGALS1* may enhance the activity of the BCR downstream PI3K/AKT pathway, thereby inhibiting tumor cell apoptosis and promoting immune evasion. Notably, in a hypoxic microenvironment, Galectin-1 secreted by cancer-associated fibroblasts (CAFs) is further upregulated, which may drive the drug-resistant phenotype by activating the VEGF (27), suggesting its potential as a target for reversing rituximab resistance.

Tissue inhibitor of metalloproteinases-1 (*TIMP1*) is a multifunctional matrix metalloproteinase inhibitor that promotes tumor cell survival and angiogenesis by binding to the STAT3 pathway (28). In anaplastic large cell lymphoma (ALCL) positive for anaplastic lymphoma kinase (ALK+), the aberrant overexpression of *TIMP1* is directly associated with persistent STAT3 phosphorylation, thereby accelerating tumor progression (29). Interestingly, in patients with DLBCL, circulating *TIMP1* levels have been identified as an independent prognostic biomarker, with high serum *TIMP1* levels indicating shorter progression-free survival (30). However, under hypoxic conditions, the expression dynamics of *TIMP1* exhibit a dual nature: it restricts tumor invasion by inhibiting *MMP-9* and reducing extracellular matrix degradation, but simultaneously promotes chemoresistance by activating the integrin β1/FAK signaling pathway. This paradoxical effect may explain the complexity of its role in hypoxia-related prognostic evaluations. However, due to the inability of this study to distinguish subtype-specific effects, the paradoxical effect could not be validated.

Annexin A1 (*ANXA1*) is a calcium-dependent phospholipid-binding protein that participates in tumorigenesis by regulating inflammatory responses and apoptosis (31). In DLBCL, siRNA-mediated knockdown of *ANXA1* significantly downregulates pro-apoptotic proteins such as Bcl-2-associated X protein (Bax) and cleaved caspase-3, while upregulating anti-apoptotic protein Bcl-2 and pro-inflammatory cytokines (e.g., TNF-α, IL-6), indicating that *ANXA1* has dual functions in promoting apoptosis and suppressing inflammation (32). Additionally, under hypoxic stress, *ANXA1* inhibits glycolytic reprogramming mediated by HIF-1α, thereby reducing the sensitivity of tumor cells to rituximab. Clinical data further support the association between low *ANXA1* expression and poor prognosis in DLBCL patients, suggesting its potential as a sensitizing target for combination immunotherapy (33).

Glycoprotein Non-Metastatic Melanoma Protein B (*GPNMB*) is a transmembrane receptor that drives tumor progression by activating dual signaling pathways, namely Wnt/β-catenin and PI3K-AKT-mTOR (34). In DLBCL, overexpression of *GPNMB* promotes nuclear translocation of β-catenin by targeting YAP1, thereby enhancing the transcription of cyclin D1 and c-Myc, which in turn accelerates tumor proliferation (35). Notably, under hypoxic conditions, *GPNMB* inhibits autophagy via an mTORC1-dependent pathway, leading to increased efflux of chemotherapeutic drugs and resistance to rituximab. Pan-cancer studies have also shown that the ability of *GPNMB* to activate the PI3K-AKT pathway is positively correlated with the metastatic potential of tumors, and inhibition of its expression significantly reduces the invasiveness of DLBCL cells (36, 37).

Cell division cycle-associated protein 7 (*CDCA7*) is a core target of MYC-dependent transcriptional regulation and is aberrantly overexpressed in MYC-rearranged diffuse large B cell lymphoma (DLBCL) (38, 39). *CDCA7* stabilizes MYC protein through AKT-mediated phosphorylation, thereby inhibiting the expression of pro-apoptotic factors such as Bim and promoting lymphoma cell transformation (40). Mechanistic studies have shown that hypoxia enhances the transcriptional activity of *CDCA7* by enabling direct binding of HIF-1α to its promoter region, forming a positive feedback loop of MYC-HIF-CDCA7 that exacerbates genomic instability. Clinical cohort analysis revealed that high expression of *CDCA7* is significantly linked to shortened overall survival in DLBCL patients, and its co-occurrence with MYC rearrangement indicates a poorer response to rituximab therapy.

Signal transduction adaptor protein 1 (*STAP1*) is a key adaptor molecule downstream of the BCR signaling pathway, regulating *STAT5* phosphorylation by recruiting SYK kinase (41). In chronic myeloid leukemia (CML), *STAP1* deficiency leads to impaired *STAT5* activity, thereby downregulating the expression of anti-apoptotic genes such as Bcl-2 and Bcl-xL (42). In the context of DLBCL, overexpression of *STAP1* enhances the sustained activation of the BCR-PI3K/AKT pathway, promoting tumor cell survival and inducing resistance to rituximab. Hypoxic microenvironments may further amplify the pro-survival effects of *STAP1*: hypoxia increases AKT phosphorylation by downregulating *PTEN* expression, which synergizes with *STAP1* to maintain STAT5 signaling, ultimately driving the expansion of drug-resistant clones.

Our RT-qPCR validation experiments revealed that some of the identified genes like *LGALS1*, *TIMP1*, *GPNMB* and *CDCA7* may be involved in hypoxia-related responses, others may not be as strongly associated with hypoxia in the context of DLBCL. These findings refine our understanding of the potential regulatory mechanisms underlying drug tolerance in hypoxic DLBCL tissues. Although our LASSO-based model identifies *LGALS1* and *STAP1* as top-ranking MS4A1 regulators, the correlative nature of transcriptomic data and the validation at transcriptional level cannot directly establish causality. To determine whether these genes exert post-transcriptional control over *MS4A1*, we will conduct CRISPR-interference knock-down of *LGALS1* and *STAP1* followed by flow-cytometric quantification of *MS4A1*, and chromatin immunoprecipitation assays to test physical occupancy of *LGALS1*/*STAP1* at the *MS4A1* promoter. Results from these experiments will clarify whether the observed statistical association reflects a mechanistic regulatory axis or an indirect co-regulation phenomenon.

Taken together, the 15 genes that make up the *MS4A1* regulator genes and PPI hub genes show differential expression in hypoxic DLBCL tissues and are thought to regulate cancer cells via the BCR and PI3K/AKT signaling pathways. These genes may serve as potential therapeutic targets and prognostic indicators for improving rituximab sensitivity and reversing drug tolerance in cancer cells, given their significant role in regulating *MS4A1* expression in hypoxic DLBCL tumors.

We predict that the regulatory mechanisms of these potential genes will provide novel perspectives on the mechanisms of drug tolerance in hypoxic DLBCL tissues. Future studies should focus on further elucidating the specific regulatory mechanisms of these genes, particularly those that exhibited hypoxic responses, and exploring their co-expression patterns and network associations. This will help validate our findings and explore their clinical implications more comprehensively.

## 5 Conclusions

In summary, an integrated bioinformatics analysis was conducted to explore hypoxia-induced rituximab resistance in DLBCL. The findings suggest that genes such as *LGALS1*, *TIMP1*, *GPNMB* and *CDCA7* possibly implicated in the BCR and PI3K-AKT signaling pathways. These genes play a crucial role in the pathophysiological mechanisms driving hypoxia-induced rituximab resistance. Our findings could provide opportunities for developing new therapeutic strategies and enhance comprehensive understanding of the mechanisms involved.

## Data Availability

Publicly available datasets were analyzed in this study. This data can be found here: https://www.ncbi.nlm.nih.gov/geo/query/acc.cgi?acc=GSE10846 https://www.ncbi.nlm.nih.gov/geo/query/acc.cgi?acc=GSE56315 https://www.ncbi.nlm.nih.gov/geo/query/acc.cgi?acc=GSE104212.

## Supplementary material

Supplementary Figure 1 Heatmap for DSGs expression levels

Supplementary Figure 2 Heatmap for HRGs expression levels
