# Supplementary figures and images for: Unveiling hypoxic regulatory networks by bioinformatics: mechanisms of hypoxia-related hub genes driving rituximab resistance and poor prognosis in DLBCL
Source: Front Oncol. 2025 Oct 20;15:1592441. doi: 10.3389/fonc.2025.1592441 (PMC12580093; doi:10.3389/fonc.2025.1592441)

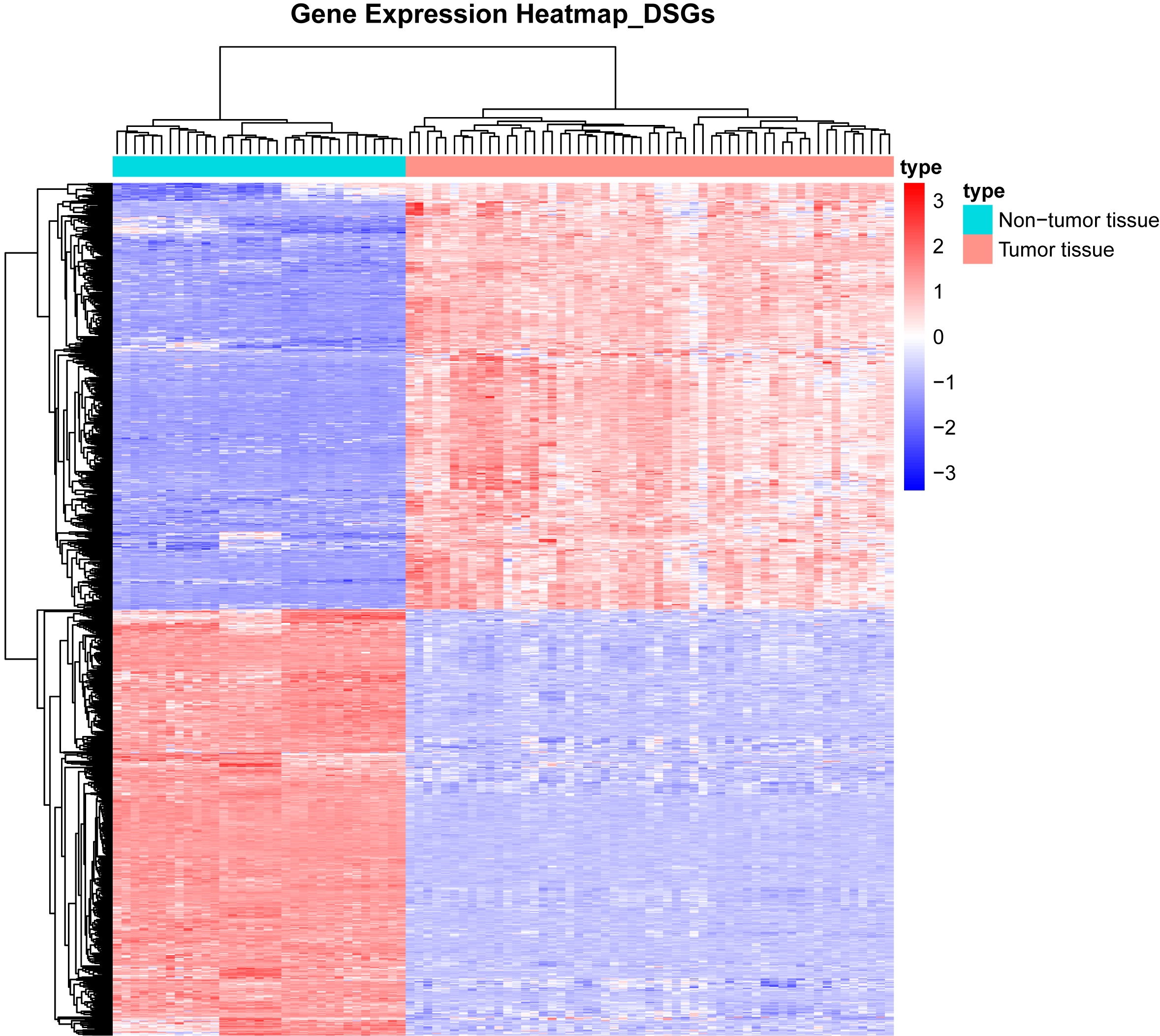

Supplement: Supplementary Figure 1 — Heatmap for DSGs expression levels [file Image1.jpeg]

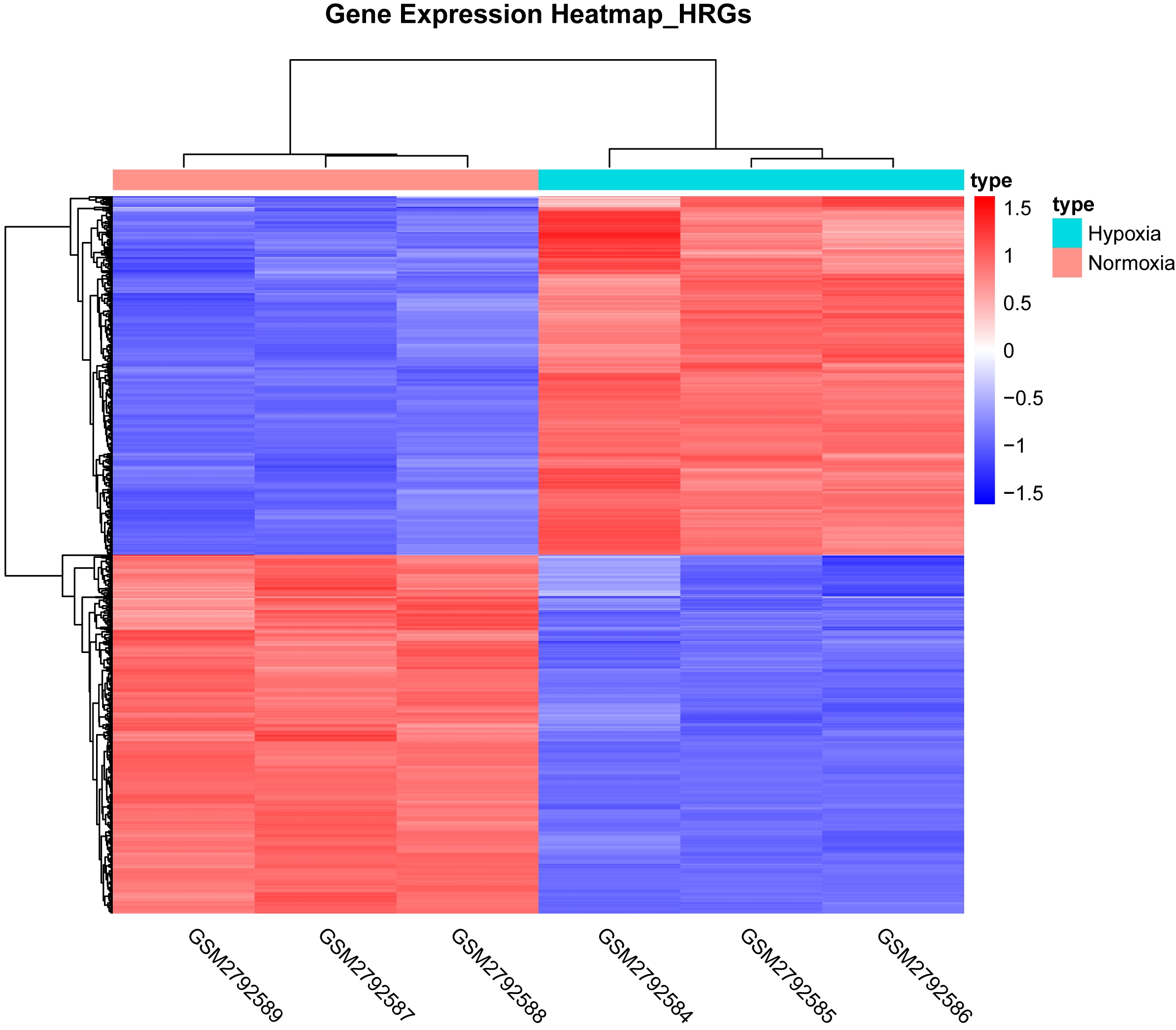

Supplement: Supplementary Figure 2 — Heatmap for HRGs expression levels [file Image2.jpeg]
